# The Roles of RANK/RANKL/OPG in Cardiac, Skeletal, and Smooth Muscles in Health and Disease

**DOI:** 10.3389/fcell.2022.903657

**Published:** 2022-05-26

**Authors:** Laetitia Marcadet, Zineb Bouredji, Anteneh Argaw, Jérôme Frenette

**Affiliations:** ^1^ Centre Hospitalier Universitaire de Québec, Centre de Recherche Du Centre Hospitalier de L’Université Laval (CHUQ-CHUL), Axe Neurosciences, Université Laval, Quebec City, QC, Canada; ^2^ Département de Réadaptation, Faculté de Médecine, Université Laval, Quebec City, QC, Canada

**Keywords:** RANK, RANKL, OPG, myokines, osteokines, heart, skeletal muscle, smooth muscle

## Abstract

Although their physiology and functions are very different, bones, skeletal and smooth muscles, as well as the heart have the same embryonic origin. Skeletal muscles and bones interact with each other to enable breathing, kinesis, and the maintenance of posture. Often, muscle and bone tissues degenerate synchronously under various conditions such as cancers, space travel, aging, prolonged bed rest, and neuromuscular diseases. In addition, bone tissue, skeletal and smooth muscles, and the heart share common signaling pathways. The RANK/RANKL/OPG pathway, which is essential for bone homeostasis, is also implicated in various physiological processes such as sarcopenia, atherosclerosis, and cardiovascular diseases. Several studies have reported bone-skeletal muscle crosstalk through the RANK/RANKL/OPG pathway. This review will summarize the current evidence indicating that the RANK/RANKL/OPG pathway is involved in muscle function. First, we will briefly discuss the role this pathway plays in bone homeostasis. Then, we will present results from various sources indicating that it plays a physiopathological role in skeletal, smooth muscle, and cardiac functions. Understanding how the RANK/RANKL/OPG pathway interferes in several physiological disorders may lead to new therapeutic approaches aimed at protecting bones and other tissues with a single treatment.

## Introduction

Muscles and bones share a common embryonic origin, the mesoderm. Both tissues develop together, and their proximity and interaction enable, among other things, kinesis, stability, and support for the body. Bones and muscles are constantly adapting to varying mechanical, physiological, and biochemical demands. Bone formation and resorption depend largely on the mechanical load resulting from gravity and muscle contractions (Brotto and Bonewald, 2015). Bone density increases rapidly during the teenage years and continues to increase into the 30s (Greenlund and Nair, 2003) in synchrony with muscle mass, which follows the same trajectory. In the elderly, physical activity and anabolic hormones such as testosterone and estrogen decrease, inducing a progressive loss of bone density and muscle mass, an important component of frailty syndrome. Cast immobilization, prolonged bed rest, microgravity, critical illness, and neuromuscular diseases can markedly accelerate the osteoporotic and sarcopenic processes (Hamrick et al., 2006; McKay and Smith, 2008; Russo, 2009; Ness and Apkon, 2014; Owen et al., 2015). Bone and skeletal muscles are thus very dynamic tissues that undergo several modifications throughout life.

Several reports over the past 10–15 years have demonstrated that the synchronicity between bones and skeletal muscle extends beyond solely mechanical loading and that osteokines and myokines released by these two tissues can potentially influence bone and skeletal muscle mass (Brotto and Bonewald, 2015; Boulanger Piette et al., 2018). We hypothesized over a decade ago that the triad composed of receptor activator of nuclear factor kappa B (RANK), its ligand (RANKL), and osteoprotegerin (OPG), an inhibitor of RANKL that plays a central role in bone remodeling and homeostasis (Yasuda, 2021), is also involved in the regulation of skeletal muscle function (Dutka et al., 2021; Yasuda, 2021). In this review, we take a closer look at the role of RANK/RANKL/OPG (RRO) in muscle function. We examine current evidence implicating the triad in cardiac, skeletal, and smooth muscle function in health and disease.

## RANK/RANKL/OPG in Bone Homeostasis and Disease

**FIGURE 1:**
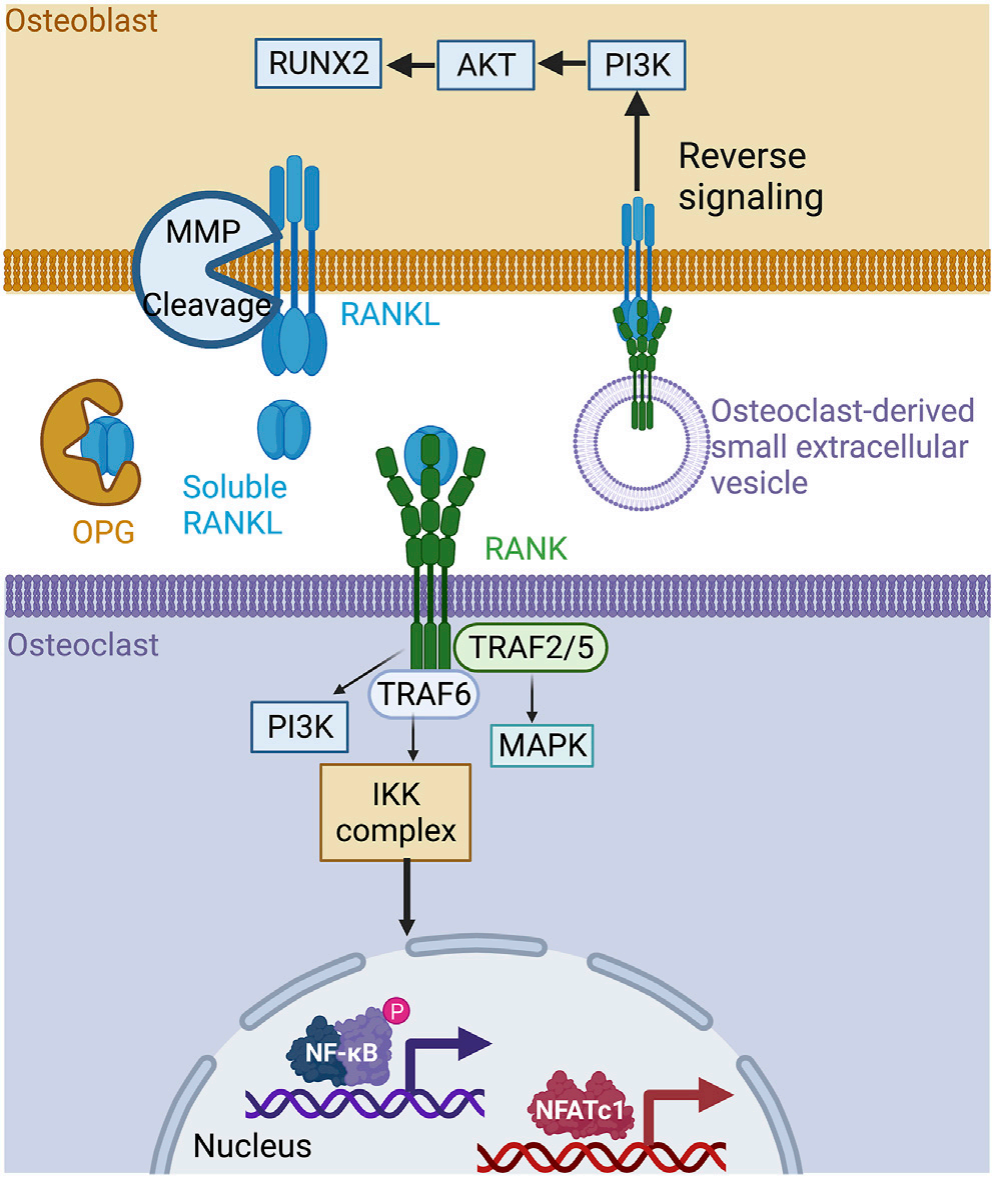
The RANK/RANKL/OPG signaling pathway in bone. RANKL is produced by osteoblasts in membrane form and is cleaved by metalloproteinases to its soluble form. The soluble form of RANKL is neutralized by circulating OPG or is bound to RANK at the osteoclast membrane, inducing a signaling cascade involving TRAF−2, −5, −6, PI3K, and MAPK, leading to the activation of the transcription factors NFATC1 and NF-κB, which are essential for bone resorption. Mature osteoclasts can also produce small extracellular RANK vesicles on their surface. These vesicles bind to the membrane form of RANKL on osteoblasts, inducing reverse signaling and promoting osteoblast differentiation. Created with BioRender.com.

**FIGURE 2:**
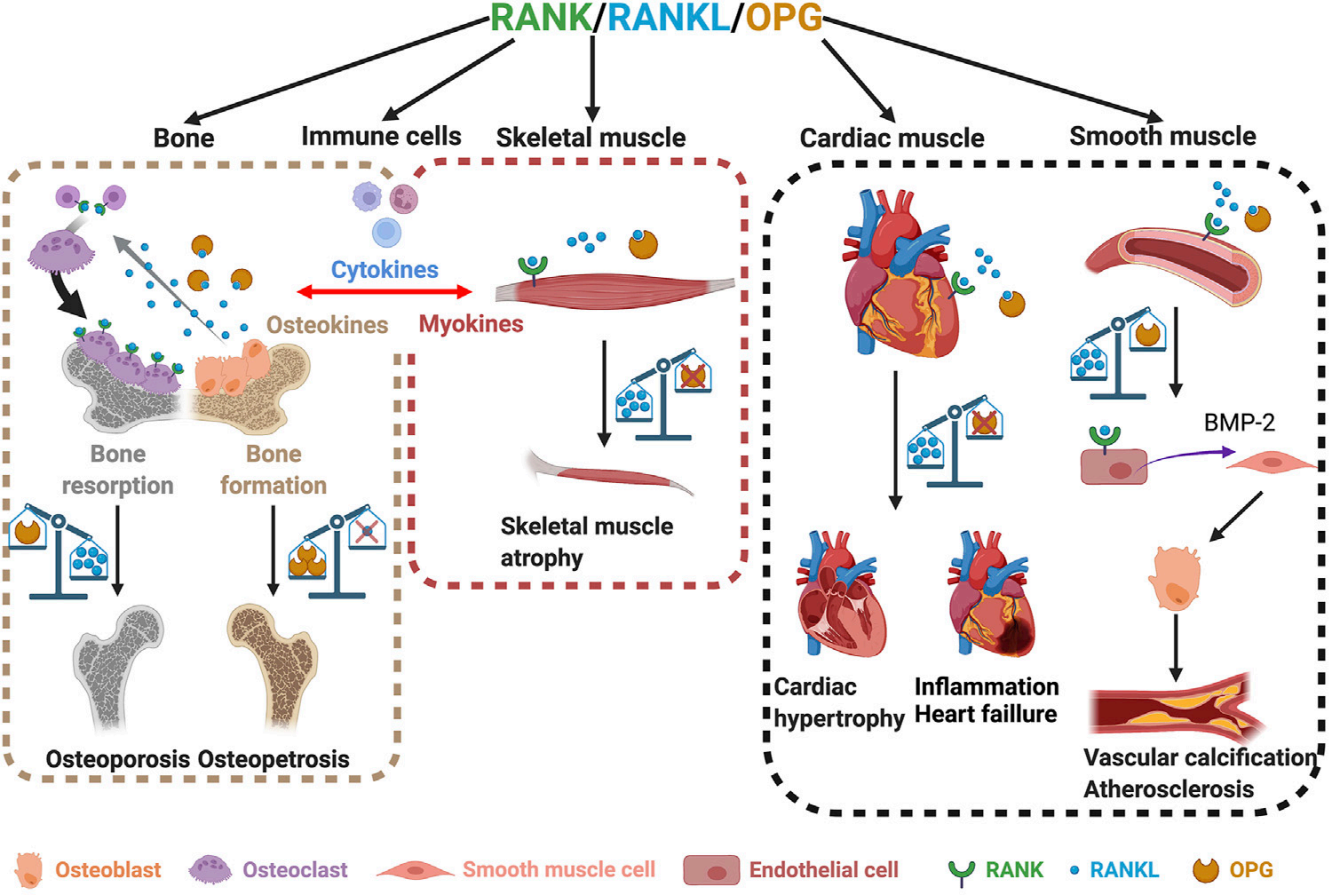
The roles of the RANK/RANKL/OPG triad in bone and skeletal, cardiac, and smooth muscles. Involvement of RANK/RANKL/OPG in bone homeostasis (left box). An imbalance between RANKL and OPG leads to either osteoporosis or osteopetrosis (left box). Skeletal muscles also express RANK, RANKL, and OPG (middle box). The overexpression of RANKL and/or the absence of OPG lead to muscle atrophy (middle box). An imbalance in the RANKL/OPG ratio also affects heart and smooth muscles and induces cardiac hypertrophy, heart failure, and vascular calcification (right box). Created with BioRender.com.

RANKL is a type II transmembrane protein expressed by the osteoblasts, osteocytes, and immune cells making up bone tissue. Structurally, RANKL has a short N-terminal intracellular tail and a larger C-terminal extracellular region (Nelson et al., 2012). Its ectodomain is cleaved to generate soluble RANKL, which is released into the extracellular milieu (Ono et al., 2020). Precursor messenger RNA (pre-mRNA) alternative splicing generates soluble and circulating RANKL (Ikeda et al., 2001). Three isomers of RANKL have been identified, including RANKL 3, a soluble protein devoid of intracellular or transmembrane domains (Ono et al., 2020; Ikeda et al., 2001). In solution, RANKL forms a homotrimer that interacts with its receptor RANK and the decoy receptor OPG (Nelson et al., 2012). RANK is a type I transmembrane protein located on osteoclast progenitors, mature osteoclasts, and immune cells (Wada et al., 2006). Osteoblast membrane-bound RANKL binds to RANK at the surface of osteoclast progenitor cells to stimulate osteoclastogenesis, bone remodeling, and calcium homeostasis (Li et al., 2000). The cytoplasmic region of RANK has no intrinsic kinase activity and requires adapter molecules for downstream signaling. Upon activation by RANKL, RANK receptors trimerize and recruit the tumor necrosis factor (TNF) associated with factor-6 (TRAF-6), an E3 ubiquitin ligase required for osteoclast differentiation (Armstrong et al., 2002). While TRAF- 2, 5, and 6 all have the ability to bind the cytoplasmic domain of RANK (Hsu et al., 1999), only TRAF-6 mutations lead to osteopetrosis, or overly dense bones, resulting from the loss of osteoclast function (Darnay et al., 1998; Lomaga et al., 1999; Kobayashi et al., 2001). RANKL/RANK binding activates downstream intracellular signaling pathways through TRAF-6, including MAPK, NF-κB, and PI3K, resulting in an increase in the expression of NFATc1, which promotes osteoclastogenesis and bone resorption (Boyce et al., 2015) (Figure 1). OPG belongs to the TNF receptor superfamily. It is secreted by osteoblasts and acts as a soluble decoy receptor of RANKL. The OPG/RANKL interaction prevents osteoclast formation and bone resorption *in vivo* (Yasuda et al., 1998). The RANKL/OPG ratio is an indicator of bone health and reflects the balance between bone formation and resorption. Unsurprisingly, RANKL/RANK expression and bone remodeling are also regulated by other cytokines, hormones, and environmental factors. For example, cytokines like interleukin-1 (IL-1 β), IL-11, and TNF-α, and hormones like 1α,25-dihydroxyvitamin D_3_, parathyroid hormone (PTH), and prostaglandin (PG) can all stimulate bone resorption by inducing the membrane expression of RANKL (Hofbauer et al., 2000; Udagawa et al., 2000). Adding to this complexity, the RANKL/RANK interaction is also implicated in bone formation. A study has reported that vesicular RANK, which is secreted from maturing osteoclasts, promotes bone formation by triggering RANKL reverse signaling and the activation of Runt-related transcription factor 2 (Runx2). This reverse signaling occurs *via* the proline-rich motif of the RANKL cytoplasmic domain (Ikebuchi et al., 2018) (Figure 1). The disruption of components of the RRO triad causes bone dysfunction and results in a pathological condition. RANKL-deficient mice exhibit a significant increase in bone mass, causing severe osteopetrosis with a lack of marrow spaces, dental eruption, and a total absence of osteoclasts. These mice show signs of growth retardation affecting several bones of the limbs, skull, and vertebrae (Kim et al., 2000). Similarly, RANK-deficient mice are resistant to bone resorption induced by TNF-α, IL-1β, calcitriol, and parathyroid hormone-related protein (PTHrP) (Li et al., 2000). In addition, osteoclast differentiation is blocked, resulting in significant osteopetrosis (Dougall et al., 1999) (Figure 2, left box). On the other hand, OPG-deficient mice develop early onset osteoporosis (Bucay et al., 1998) (Figure 2, left box). In humans, genetic mutations affecting the RANK, RANKL, and OPG genes are associated with familial forms of bone abnormalities. Mutations in the signal peptide region of RANK have been linked to familial Paget disease (Hughes et al., 2000) and to forms of singular anomalies such as expansile skeletal hyperphosphatasia. This anomaly, which has been observed in a daughter and her mother, is caused by a 15-base pair duplication in the RANK gene and is characterized by accelerated bone remodeling that results in bone, dental, and metabolic dysfunctions (Whyte and Hughes, 2002). Mutations in the gene encoding OPG cause idiopathic hyperphosphatasia, an autosomal recessive bone disease characterized by deformities of long bones, kyphosis, and acetabular protrusion (Cundy et al., 2002). The severity of the condition increases during adolescence. Polymorphisms in the OPG gene are also associated with osteoporotic fractures (Langdahl et al., 2002). A recent study showed that RANKL is involved in the pathophysiology of fibrous dysplasia of bone (FD), a genetic disease affecting the skeleton where postnatal skeletal stem cells acquire a fibroblastic phenotype and proliferate, replacing bone marrow resident cells and causing bone demineralization, an increase in osteoclast density, and, consequently, the dysregulation of osteoclastogenesis (de Castro et al., 2019). Patients with FD exhibit a 12-fold increase in serum RANKL/OPG ratios, which is significantly correlated with the FD burden (de Castro et al., 2019). The RANKL/RANK signaling pathway is also implicated in osteoporosis associated with estrogen deficiency in early postmenopausal women. Increased cell surface expression of RANKL by bone marrow cells has been observed, which directly correlates with increased osteoclast formation and bone resorption in aging women (Eghbali-Fatourechi et al., 2003). Targeting the RRO signaling pathway remains a promising therapeutic strategy to reduce bone resorption and ultimately bone loss. Denosumab, a human monoclonal antibody targeting RANKL, has received FDA approval for treating osteoporosis in men and postmenopausal women and minimizing bone loss associated with metastases in patients with advanced solid tumors (Cummings et al., 2009; Lacey et al., 2012). Denosumab has recently been shown to be more effective in treating glucocorticoid (GC)-induced osteoporosis than bisphosphonates (Yanbeiy and Hansen, 2019). RRO has also been reported to be involved in osteoclast formation and bone resorption in rheumatoid arthritis (RA), a systemic autoimmune disease (Tanaka and Tanaka, 2021). A recent case-controlled study and meta-analysis has indicated that the RANKL gene polymorphism increases the risk for RA. However, RANK and OPG gene locus polymorphisms are not associated risk factors (Yang et al., 2019). A recent study has shown that RANKL serum concentrations are highest in RA associated with periodontal disease (PD) and that a denosumab treatment suppresses the progression of RA in a randomized controlled trial (Panezai et al., 2018; Tanaka and Tanaka, 2021). Lastly, numerous studies have shown that the RRO pathway is involved in bone metastases (Ono et al., 2020; Okamoto, 2021) and that the neutralization of RANKL appears to have a beneficial effect on bone health. Clinical trials are currently investigating whether denosumab can be used to treat different cancers (Raje et al., 2018). However, a recent international randomized controlled trial with denosumab failed to improve disease-related outcomes for women with high-risk early breast cancer (Coleman et al., 2020). The RRO pathway is thus essential for the regulation of bone homeostasis, while its deregulation may be involved in bone, autoimmune, and cancerous diseases.

## RANK/RANKL/OPG in Cardiac and Smooth Muscles

It is now well established that the role of the triad RRO goes beyond bone homeostasis, mammary gland development, and immunity. Interestingly, studies of heart failure in rodents have shown a high and persistent expression of the RANK, RANKL, and OPG genes in the ischemic and nonischemic areas of the heart (Ueland et al., 2005; Slavic et al., 2018). Conversely, the selective inhibition of RANKL in hematopoietic cells is sufficient to reduce the expression of pro-inflammatory cytokine IL1-ß and maintain post-ischemic cardiac function (Slavic et al., 2018). Furthermore, intravenous post-infarction anti-RANKL treatments in C57BL/6 mice reduce infarct size and cardiac neutrophil infiltration (Carbone et al., 2016). Likewise, OPG-deficient mice exhibit cardiac hypertrophy and myocardial contractile dysfunction at as early as 2.5 months of age (Hao et al., 2016). These morphological changes are accompanied by an increase in the number of apoptotic cells and the activation of TNF-related apoptosis-inducing ligand (TRAIL). A 28-day treatment with exogenous OPG partially rescued left ventricular structure and function in OPG-deficient mice (Hao et al., 2016). The inhibition of TRAIL decreases myocardial infarction by preventing cardiac cell death and inflammation in rats, pigs, and monkeys (Wang et al., 2020). Mechanistically, TRAIL induces the death of recruited leukocytes and activated cardiomyocytes, causing cardiac injury (Wang et al., 2020). In a model of pressure overload, a marked increase in RANKL expression has been observed in hypertrophying myocardium while *in vitro* RANKL stimulates the expression of TNFα, IL-1α, and IL-1β *via* the TRAF6-NF-κB signaling pathway in neonatal cardiomyocytes (Ock et al., 2012). These results from animal models have been confirmed in patients with heart failure who also exhibit increased RANK, RANKL, and OPG protein concentrations, suggesting that they are involved in the development of heart failure (Ueland et al., 2005). As such, the RRO pathway is thought to be involved in cardiac remodeling following immunoinflammatory myocardial diseases or during chronic heart failure (Liu et al., 2008) (Figure 2, right box).

Smooth muscles form the dense layers of many tissues, including blood vessels and hollow organs. Unlike skeletal and cardiac muscles, smooth muscles do not have transverse striations. Vascular smooth muscle cells (VSMCs) are involved in vascular tone, and their contractile function is not under voluntary control (Iyemere et al., 2006). VSMCs participate in vascular and valve calcification and share common characteristics with bone remodeling and metabolism (Kawakami et al., 2016). Many bone proteins are expressed in calcified vessel plaques, including bone morphogenetic protein-2 (BMP-2), an osteogenic differentiation factor capable of differentiating VSMCs into osteoblast-like cells (Kawakami et al., 2016). Under basal conditions human aortic smooth muscle cells (HASMCs) produce OPG whose expression increases with inflammatory stimuli (Davenport et al., 2018). OPG expression is inhibited when exogenous RANKL is added to the microenvironment of HASMCs (Davenport et al., 2018). RANKL acts also on human aortic endothelial cells (HAECs), increasing the release of BMP-2 from HAECs, which, in turn, can potentially stimulate osteoblast-like cell activity in HASMCs (Davenport et al., 2016). OPG is naturally present in blood vessels. The presence of RANKL inhibits OPG secretion by HASMCs and disrupts the RANKL/OPG ratio, promoting vascular calcification (Davenport et al., 2016; Davenport et al., 2018). Like OPG, matrix Gla protein (MGP) is an inhibitor of calcification that prevents the precipitation of calcium salts. Estrogen inhibits RANKL-mediated BMP-2 release and increases MGP expression (Osako et al., 2010). This supports the observation that aging women with estrogen deficiency are more at risk of both cardiovascular diseases and osteoporosis after menopause (Lampropoulos et al., 2012; Sprini et al., 2014). The RRO pathway thus plays an active role in angiogenesis, pathological inflammation, cell survival, and VSMC calcification (Rochette et al., 2019), pointing to potential cross-talk between blood vessels and bones (Figure 2, right box).

A recent review presented current knowledge on the roles of the different elements of the RRO triad in heart failure and cardiovascular diseases (Dutka et al., 2021). OPG plays an important role in the cardiovascular system through its interactions with endothelial and smooth muscle cells and its ligands, TRAIL and RANKL. Circulating OPG concentrations have been proposed as a biochemical marker for assessing the risk of cardiovascular complications in osteoporotic patients (Barbu et al., 2017). Elevated OPG concentrations in serum may play a protective role in the early stages of cardiovascular pathology. However, the maintenance of high OPG concentrations may have deleterious effects on the vascular system by participating in atherogenesis and vascular injury (Dutka et al., 2021). On the other hand, elevated serum RANKL concentrations may increase the risk of cardiovascular diseases (Kiechl et al., 2007). To go further in highlighting the crosstalk between bones and vessels, few studies have also reported the interaction between the RRO triad and blood coagulation factors. An *in vitro* model showed that differentiated osteoclasts would release RANKL, activating the extrinsic coagulation pathway and the conversion of prothrombin to thrombin (Karlström et al., 2010). Another study showed that thrombin receptor deficiency leads to a decrease in RANKL/OPG ratio which was associated with a high bone density phenotype (Tudpor et al., 2015). Conversely, a significant increase in the RANKL/OPG ratio and signs of osteoporosis were noticed in a mouse model of hemophilia (Yen et al., 2022). Moreover, OPG may play a role in regulating thrombus formation by binding to Von Willebrand factor (VWF), an essential factor for platelet adhesion (Wohner et al., 2022). Although significant progress has been made in the field, the molecular mechanisms by which RANK, RANKL, and OPG modulate cardiovascular diseases, heart failure and to some extent hemostasis remain unclear and need further investigation.

## The RANK/RANKL/OPG Pathway in Dystrophic Skeletal Muscle, Inflammation, and Repair

Like bone cells and cardiac and smooth muscles, skeletal muscles also express the RRO triad (Baud’huin et al., 2013; Dufresne et al., 2018; Dufresne et al., 2016). A study on healthy human volunteers has shown that intense exercise-induced muscle damage increases serum OPG and decreases RANKL concentrations, suggesting that they are involved in muscle inflammation and repair processes in response to damage (Philippou et al., 2009). Changes in circulating OPG and RANKL concentrations are correlated with the distance traveled by runners, implying that the positive effects of long distance running on skeletal mass may, in part, be mediated by OPG/RANKL (Ziegler et al., 2005). Previous work from our laboratory has also shown that muscle-specific RANK deletion modulates the regulation of Ca^2+^ storage and sarco-endoplasmic reticulum Ca^2+^-ATPase (SERCA) activity in skeletal muscle (Dufresne et al., 2016). In the context of muscle disease, dystrophic *mdx* muscles exhibit significantly higher concentrations of RANK than wild-type muscles, suggesting that RANK is involved in muscular dystrophy (Dufresne et al., 2018; Guiraud et al., 2017). We have shown that daily injections of OPG-Fc, restore the function of the *extensor digitorum longus* (EDL) muscle in young dystrophic *mdx* mice (Dufresne et al., 2015). The inhibition of the RANKL/RANK interaction with an antibody specifically targeting RANKL also improves muscle strength in *mdx* mice (Hamoudi et al., 2019). We used a severe myotoxic agent to induce skeletal muscle injury and showed that the daily administration of the recombinant full-length OPG-Fc (FL-OPG-Fc) protein improves muscle strength, regeneration, and repair (Bouredji et al., 2021). On the other hand, genetic deletion of OPG in mice results in osteoporosis at as early as 3 months of age, with progressive muscle atrophy by 5 months (Hamoudi et al., 2020) (Figure 2, middle box). The concentration of circulating RANKL increases 20-fold in these OPG-deficient mice (Hamoudi et al., 2020). We showed that a 2-month treatment with anti-RANKL significantly improves muscle strength and reduces osteoporosis in OPG-deficient mice (Hamoudi et al., 2020). Conversely, mice that overexpress RANKL or that lack the myogenic factor peroxisome proliferator-activated receptor beta (Pparb) exhibit lower maximal strength and velocity and reduced muscle mass (Bonnet et al., 2019) (Figure 2, middle box). Treatments with truncated OPG-Fc (TR-OPG-Fc) or denosumab increase the muscle mass and strength of these two mouse models (Bonnet et al., 2019). They used a translational experimental approach to show that postmenopausal women with osteoporosis who were treated with denosumab for 3 years exhibit improved lean appendicular mass and grip strength while bisphosphonates have no effect (Bonnet et al., 2019). Moreover, other conditions such as chronic cigarette usage or chronic obstructive pulmonary disease (COPD) correlate with high serum RANKL concentrations (Bai et al., 2011; Nogueira and Breen, 2021) and low BMD and muscle dysfunction (Nogueira and Breen, 2021). A 6-month chronic exposure to cigarette smoking increases RANK and RANKL concentrations in skeletal muscle fibers (Xiong et al., 2021). Interestingly, the neutralization of RANKL restores muscle strength and function in mice exposed to tobacco smoke particles (Xiong et al., 2021). Furthermore, the exposure of muscle cells to tobacco smoke particles *in vitro* causes an increase in the expression of RANKL/RANK, which is responsible for the inflammation and atrophy of muscle fibers (Xiong et al., 2021). Lastly, mice with non-metastatic ovarian cancer exhibit cachexia associated with high RANKL concentrations, while an anti-RANKL treatment reduces bone loss and improves muscle function (Pin et al., 2021). Taken together, this evidence shows that RANK and RANKL are involved in skeletal muscle dysfunction while their inhibitors, anti-RANKL, TR-OPG-Fc, and FL-OPG-Fc, provide beneficial effects and may serve as a potential therapeutic approach in the future.

## Crosstalk Between Bone and Skeletal Muscle Through the RANK/RANKL/OPG Pathway

Physical activity contributes to cytokine release and bone development and remodeling (Tobeiha et al., 2020). For example, circulating concentrations of OPG and RANKL increase significantly immediately following high intensity aerobic exercise, suggesting that bone remodeling mediators have been activated (Mezil et al., 2015). Furthermore, a one-year training program significantly increases OPG concentrations. This has been associated with a significant reduction in bone loss in postmenopausal women compared to sedentary controls (Bergström et al., 2012). Conversely, mechanical unloading and estrogen deficiency reduce serum OPG concentrations compared with menstruating women who exercise, which provides support for the positive osteogenic effect of OPG and exercise (West et al., 2009). Recent discoveries have revealed the importance of mutual cross-talk between muscle-bone through the release of myokines and osteokines. For instance, transforming growth factor (TGF-β), which is mainly produced by osteocytes and is stored in the bone matrix (Xu et al., 2018), plays a direct role in skeletal muscle weakness in pathological conditions. The increase in circulating TGF-β following metastasis-induced bone destruction increases the oxidation of muscle proteins and Ca^2+^ receptors through the upregulation of NADPH oxidase 4 (Nox4) and contributes to muscle weakness (Waning et al., 2015). It has been shown that circulating IL-6, the first identified myokine, increases 100-fold during exercise (Pedersen and Febbraio, 2008), sending a signal to osteoblasts to favor osteoclast differentiation and the release of bioactive osteocalcin, an anabolic hormone that plays an essential role in bone and muscle mass and muscle performance (Mera et al., 2016; Chowdhury et al., 2020). IL-6 also increases the expression of RANKL, which is important for the release of bioactive osteocalcin (Chowdhury et al., 2020). Additionally, IL-6 has been reported to induce the expression of IL-10, another anti-inflammatory myokine (Steensberg et al., 2003; Santos et al., 2020). The deletion of IL-10 in mice accelerates bone resorption and has an impact on bone homeostasis (Dresner-Pollak et al., 2004). The stimulation of bone cells *in vitro* with IL-10 increase the expression of OPG and decreases the expression of RANKL (Liu et al., 2006) while the deletion of IL-10 in mice accelerates bone resorption, suggesting that IL-10 myokine may influence bone by modulating the RRO pathway. In the same vein, the administration of irisin, a myokine released from skeletal muscles after physical exercise, attenuates the negative effect of unloading on OPG, thus maintaining the equilibrium of RANKL/OPG ratio in unloaded mice (Colaianni et al., 2017). Myostatin, a negative regulator of muscle mass is another myokine that promotes the expression of several bone regulators, including RANKL, in osteolytic cell cultures (Qin et al., 2017). Mounting evidence has thus confirmed the existence of bidirectional and mutual molecular crosstalk between bone and skeletal muscle that, to some extent, directly or indirectly impacts the expression of RRO (Figure 2, left and middle boxes).

## Concluding Remarks and Future Directions

This mini review focuses on the multifaceted aspects of the RRO pathway. Although the RRO triad is the most important regulator of bone homeostasis, cumulative evidence has elegantly demonstrated that it also plays a role in skeletal, smooth, and cardiac muscles. Targeting similar pathways that regulate different tissues is undoubtedly a valuable strategy for addressing pathological and aging conditions with multiple comorbidities. While the RRO signaling cascade is well defined in bone cells, the cellular and molecular mechanisms by which RRO regulates muscle cell function is far from being well understood. RANKL is clearly associated with the activation of the inflammatory-atrophic and inflammatory-hypertrophic pathways in skeletal muscle and the heart, respectively. Future research directions should focus on investigating the clinical relevance of neutralizing RANKL in muscular and cardiac disorders such as dystrophic diseases, aging, and cancer-related cachexia. As for OPG, a clear understanding of the involvement of each of its three domains, that is, the RANKL-, TRAIL- and heparin-binding domains, is a prerequisite for deciphering the contribution of RRO in skeletal, smooth, and cardiac muscles.
